# The Role of MRI in Breast Cancer and Breast Conservation Therapy

**DOI:** 10.3390/cancers16112122

**Published:** 2024-06-01

**Authors:** Iman Washington, Russell F. Palm, Julia White, Stephen A. Rosenberg, Dana Ataya

**Affiliations:** 1 Department of Radiation Oncology, H. Lee Moffitt Cancer Center & Research Institute, 12902 USF Magnolia Drive, Tampa, FL 33612, USA; russell.palm@moffitt.org; 2 Department of Radiation Oncology, The University of Kansas Medical Center, 4001 Rainbow Blvd, Kansas City, KS 66160, USA; jwhite22@kumc.edu; 3 Department of Radiation Therapy, H. Lee Moffitt Cancer Center & Research Institute, 12902 USF Magnolia Drive, Tampa, FL 33612, USA; stephen.rosenberg@moffitt.org; 4 Department of Diagnostic Imaging and Interventional Radiology, H. Lee Moffitt Cancer Center & Research Institute, 10920 N. McKinley Drive, Tampa, FL 33612, USA; dana.ataya@moffitt.org

**Keywords:** breast MRI, MRI-guided breast radiotherapy, radiation treatment planning

## Abstract

**Simple Summary:**

Breast MRI is a valuable imaging modality that plays a critical role in the detection, diagnosis, and treatment of breast cancer. The superior sensitivity of breast MRI allows for more accurate evaluation of the extent of disease for breast cancer, which is important in an era where de-escalation of treatment is being considered and offered. In this review, we provided a detailed overview of breast MRI and its clinical utility in the management of breast cancer as a foundation for its use in the field of radiation oncology. Specifically, we describe breast MRI technique, anatomy, common breast MR imaging findings, and the role of breast MRI in assessing extent of disease and tumor response to neoadjuvant therapy. There has been increased interest in the use of MRI during radiotherapy treatment planning and delivery for patients with early-stage breast cancer as it may allow for more personalized radiotherapy.

**Abstract:**

Contrast-enhanced breast MRI has an established role in aiding in the detection, evaluation, and management of breast cancer. This article discusses MRI sequences, the clinical utility of MRI, and how MRI has been evaluated for use in breast radiotherapy treatment planning. We highlight the contribution of MRI in the decision-making regarding selecting appropriate candidates for breast conservation therapy and review the emerging role of MRI-guided breast radiotherapy.

## 1. Introduction

### 1.1. Breast Cancer Overview

Breast cancer is the most commonly diagnosed cancer and the second leading cause of cancer death among women in the United States. In 2024, an estimated 310,720 new cases of invasive breast cancer will be diagnosed, and approximately 42,250 women will die of the disease [1]. An important strategy in reducing breast cancer mortality is breast cancer screening [2]. When breast cancer is diagnosed, accurate staging is essential in selecting optimal treatment strategies and techniques. The addition of contrast-enhanced breast MRI in appropriate situations can aid in the further detection, evaluation, and management of breast cancer. 

### 1.2. Diagnostic Value of Breast MRI

Since the introduction of breast MRI in the 1980s, multiple studies have evaluated its sensitivity and cancer detection rate compared to mammography alone. The average sensitivity of digital mammography alone is approximately 80–90% in nondense breasts and approximately 50–60% in dense breasts [3,4]. In comparison, breast MRI has a sensitivity of 90–100% irrespective of breast density [5,6,7,8,9,10]. 

Multiple trials have demonstrated that breast MRI detects cancers that are mammographically, sonographically, and clinically occult [4,5,7,8,9,10,11,12,13,14,15,16]. The high incremental cancer detection rate seen with breast MRI is observed irrespective of breast density and menopausal status [17]. Furthermore, while breast MRI has a lower perceived positive predictive value (PPV), more recent published studies demonstrate the PPV of breast MRI to be similar to that of mammography [18,19]. Importantly, breast MRI has also been shown to preferentially detect intermediate and high-grade cancers, which are more likely to be biologically significant [20,21]. While the routine use of breast MRI for preoperative staging remains controversial given a lack of data, demonstrating that it leads to definitive improvement in patient outcomes [22,23], the superior diagnostic accuracy of breast MRI has resulted in the rapid adoption of breast MRI into clinical practice (Table 1).

## 2. Breast MRI Technique and Interpretation 

### 2.1. Breast MRI Protocol and Positioning

**Figure 1:**
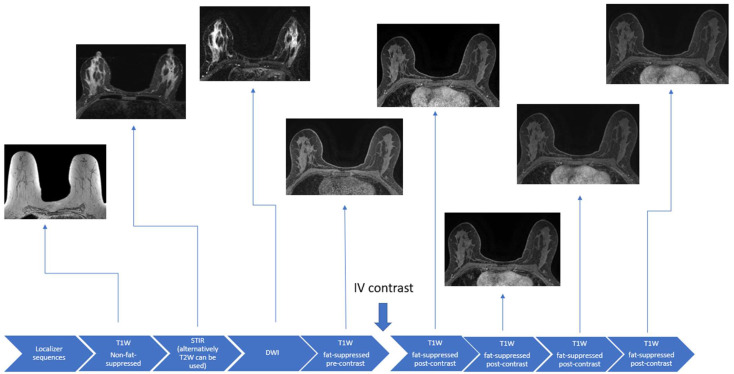
Schematic example of a full diagnostic breast MRI protocol.

During the breast MRI examination, patients lie prone with the breasts suspended in a dedicated radiofrequency breast coil. Not only does this position allow for the optimized evaluation of the breast tissues, but it can minimize artifacts due to respiratory and cardiac motion [24,25]. Contrast administration is critical in the evaluation of the breast parenchyma for diagnosis and the extent of breast cancer but is not required for exams carried out to solely assess breast implant integrity. Conventional breast MRI sequences include T1-weighted, T2-weighted, or Short Tau Inversion Recovery (STIR), and diffusion-weighted imaging (DWI) sequences (Figure 1).

### 2.2. Primary Sequences for Breast MRI

#### 2.2.1. T1-Weighted Sequences 

**Figure 2:**
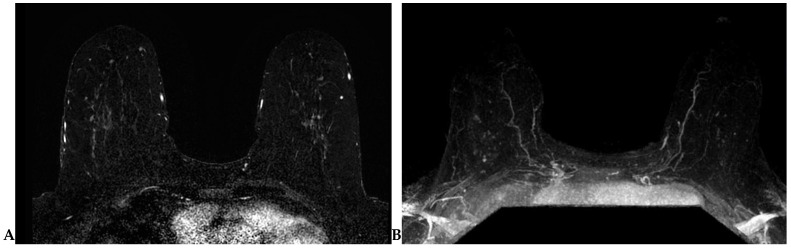
(**A**) Axial subtraction image. The pre-contrast T1W sequence is “subtracted” from each post-contrast T1W sequence to generate the subtraction images. (**B**) Axial maximum-intensity projection (MIP). A MIP can be generated from the subtraction images.

Lesion detection is largely performed on T1-weighted dynamic contrast-enhanced (DCE) sequence images. Prior to DCE sequences, a T1-weighted non-fat-suppressed sequence is typically acquired. Subsequently, pre- and post-contrast T1-weighted fat-suppressed dynamic contrast-enhanced (DCE) sequences are obtained at multiple time points. The creation of subtraction images—a post-processing technique whereby the pre-contrast sequence is “subtracted” from the post-contrast sequences—is required for DCE imaging obtained without fat suppression (Figure 2A). A maximum-intensity projection (MIP) can also be generated from these subtraction images (Figure 2B) and can assist in efficient lesion detection [26]. It is important to note that artifacts (motion, chemical shift, poor fat suppression) may degrade these post-processed sequences. 

#### 2.2.2. T2-Weighted or STIR Sequences 

The standard breast MRI protocol includes a fluid-sensitive sequence, most commonly either an axial T2-weighted (with or without fat suppression) or a short-tau inversion recovery (STIR) sequence. Although some cancers may demonstrate high signal intensity on fluid-sensitive sequences (necrotic cancers, mucinous carcinomas, and metaplastic carcinomas), most masses with high signal intensity on a fluid-sensitive sequence are benign (cysts, seromas, lymph nodes, and fibroadenomas). Studies have demonstrated that fluid-sensitive imaging increases the specificity in distinguishing benign and malignant lesions [27,28,29,30,31,32]. 

#### 2.2.3. Diffusion Weighted Imaging (DWI) 

DWI supplements DCE imaging to improve specificity while maintaining similar rates of sensitivity [33]. Although there is increasing use of DWI, it still remains an area of study. DWI offers lower spatial resolution when compared to the DCE-MR sequences. A small malignancy may not be noticeable on DWI without the corresponding DCE-MR images for correlation. DWI sequences are also more prone to MR artifacts. A consensus statement published by the EUSOBI International Breast Diffusion-Weighted Imaging working group [34] offers a basis for the standardization of DWI and is an important step in promoting DWI in clinical practice and garnering wider application and acceptance.

### 2.3. Breast Anatomy with MRI

**Figure 3:**
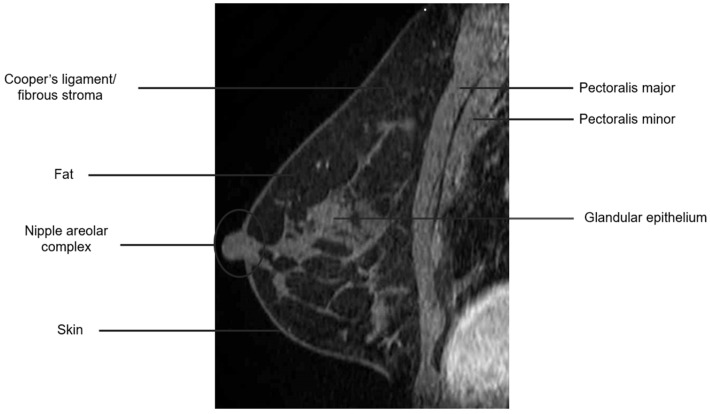
Anatomy of the breast on a sagittal T1W fat-suppressed post-contrast breast MRI.

**Figure 4:**
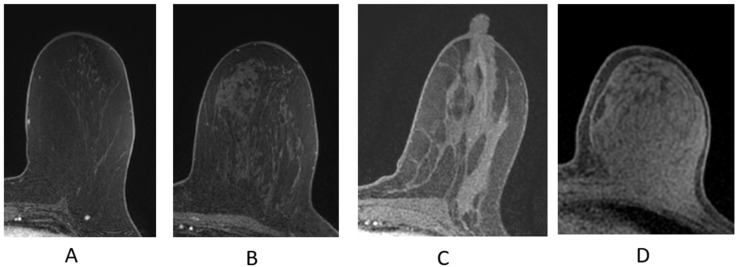
Fibroglandular tissue (FGT) depicted n axial T1W fat-suppressed breast MRI. (**A**) Almost entirely fat; (**B**) scattered fibroglandular tissue; (**C**) heterogeneous fibroglandular tissue; (**D**) extreme fibroglanduar tissue.

The breast is composed of glandular epithelium, fibrous stroma, and fat. The glandular epithelium in the breast consists of two main building blocks: ducts and lobules. Together, the ducts and lobules form ductal-lobular units, which coalesce to form approximately 15–20 lobes in a breast. Each lobe leads to a duct that widens to form a lactiferous sinus under the nipple–areolar complex prior to exiting the nipple. The fibrous stroma—frequently referred to as Cooper’s ligaments—is composed of bands of connective tissue that traverse the breast and insert into the dermis. These basic structures are seen at breast MRI (Figure 3). The amount of fibroglandular tissue (FGT) is evaluated on T1-weighted (either on the fat-saturated or non-fat-saturated) breast MR imaging sequences (Figure 4) [35]. 

**Figure 5:**
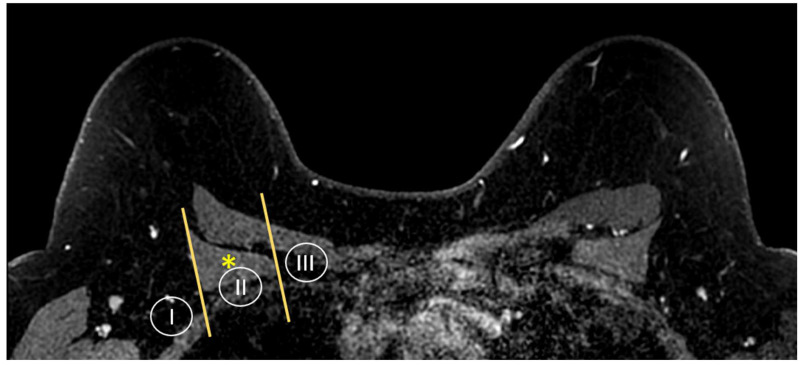
Anatomy of the axillary nodal stations on axial T1W fat-suppressed post-contrast breast MRI. Levels are defined by the relationship of the lymph node to the pectoralis minor muscle (asterisk).

**Figure 6:**
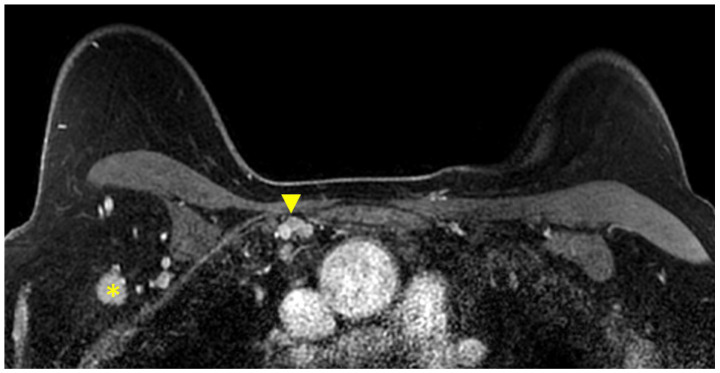
Axial T1W fat-suppressed post-contrast breast MRI provides a global view of the axillary and internal mammary nodal basins. In this case, level I axillary adenopathy (asterisk) and internal mammary adenopathy (arrowhead) are present.

MR imaging is also able to provide a global view of the axillary and internal mammary nodal stations (Figure 5 and Figure 6). Axillary lymph nodes are divided into levels I, II, and III. The levels are defined by the relationship of the lymph node to the pectoralis minor muscle. Level I lymph nodes are inferolateral to the pectoralis minor; level II lymph nodes are posterior to and between the lateral and medial borders of the pectoralis minor; and level III lymph nodes are superomedial to the pectoralis minor [36]. Internal mammary lymph nodes (IMLN) are typically visualized in the first through sixth intercostal spaces. 

### 2.4. Findings in the Breast on MRI

#### 2.4.1. Focus 

**Figure 7:**
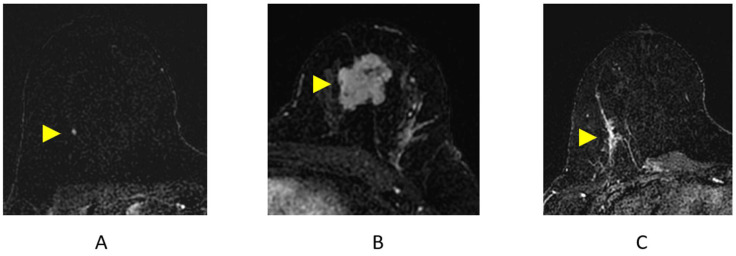
Breast MRI Findings on axial post-contrast subtraction breast MRI (arrowheads). (**A**) Focus; (**B**) mass; (**C**) non-mass enhancement (NME).

A focus is a small “dot” of enhancement that is so small, the shape and margins cannot be further characterized (Figure 7A) [35]. In clinical practice, the difference between a focus and a small subcentimeter mass is judged by the interpreting radiologist. Almost all foci identified at breast MRI are benign. In rare cases, a focus may represent an early malignancy [37,38]. 

#### 2.4.2. Mass (Figure 7B)

In contrast to a focus, a mass is large enough for shape, margin, and internal enhancement characteristics to be depicted [35]. A spiculated margin has the highest likelihood of malignancy—with a PPV of 84–91%—compared to other shape and margin descriptors [38,39,40]. 

#### 2.4.3. Non-Mass Enhancement (NME) 

NME is a term used to describe an area of enhancement distinct from background parenchymal enhancement (BPE) that is not a space-occupying mass (Figure 7C). NME typically conforms to the FGT planes and may not have a pre-contrast correlate on the T2-weighted/STIR MRI sequences [41]. NME can be further characterized by internal enhancement patterns and distribution inside the breast. For example, a segmental or linear distribution of NME carries a higher likelihood of malignancy [38,42,43,44,45]. Studies have also demonstrated that a clustered ring internal enhancement pattern has a 77–100% PPV for malignancy [43,44,45] and that clumped internal enhancement has an 88% PPV for malignancy [43]. In clinical practice, ductal carcinoma in situ (DCIS) and diffuse invasive breast cancers (particularly lobular cancers) are the most common malignant causes of NME [41].

#### 2.4.4. Background Parenchymal Enhancement (BPE)

**Figure 8:**
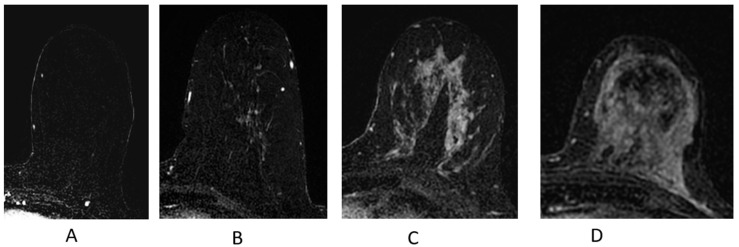
Background parenchymal enhancement (BPE). (**A**) Minimal; (**B**) mild; (**C**) moderate; (**D**) marked.

Normal FGT can enhance on breast MRI and is termed BPE. BPE should be assessed on the first post-contrast sequence on breast MRI (Figure 8). When BPE is bilateral and symmetric, the likelihood of a false-positive interpretation is low [46]. However, when BPE presents in an asymmetric focal or regional fashion, it may be difficult to differentiate from NME [41,46], and follow-up or biopsy may be recommended. Additionally, a clinical history of whole-breast radiation therapy can explain asymmetric BPE, as the irradiated breast will have significantly less BPE compared to the non-treated breast [41,46,47]. Several studies performed in the diagnostic setting have shown that BPE may impact the accuracy in the extent of disease staging in newly diagnosed breast cancer patients, particularly in cancers presenting as NME [48,49]. 

#### 2.4.5. Kinetic Curve Assessment

After an enhancing breast finding has been identified and characterized, the evaluation of lesion-enhancement kinetics is performed. The assessment of the kinetic curves of a breast lesion provides insight into pathophysiology by depicting the enhancement pattern of a lesion over time following contrast administration. These curves reflect the underlying vascular properties and microenvironment of a lesion. A classically suspicious kinetic curve includes early rapid enhancement with delayed washout, as malignant lesions tend to have increased angiogenesis, vascular permeability, and enlarged interstitial spaces, leading to the rapid uptake and washout of the contrast agent. However, the characterization of breast findings on the basis of kinetic assessment alone is not recommended due to the variability of kinetic curves in both benign and malignant pathologies. Morphologic characteristics of the breast finding should take precedence over the kinetic features. Indeed, when both morphologic and kinetic features are combined, the specificity of breast MRI interpretation improves [50]. 

#### 2.4.6. Identifying Cancers 

**Figure 9:**
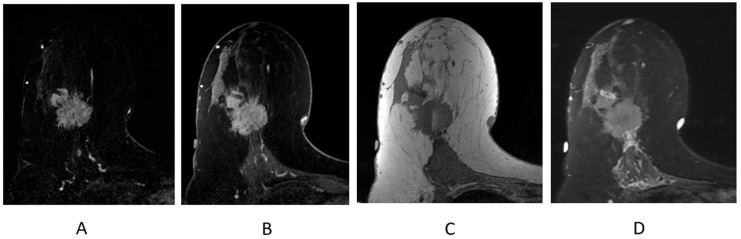
Invasive ductal carcinoma presenting as an irregular mass on breast MRI. (**A**) Axial post-contrast subtraction, first time point; (**B**) axial post-contrast T1-weighted fat-suppressed sequence; (**C**) axial T1-weighted non-fat-suppressed sequence; (**D**) axial STIR sequence.

The majority of enhancing breast cancers will be conspicuous on the first post-contrast T1-weighted sequence, particularly on the post-processed subtraction image (Figure 9A). The “source” images—the post-contrast T1-weighted fat-suppressed sequence—will demonstrate the enhancing cancer and provide information on the parenchymal contour and nearby anatomic landmarks (Figure 9B); these are the best sequences in the evaluation and contouring of a breast malignancy. 

#### 2.4.7. Identifying Lymph Nodes 

**Figure 10:**
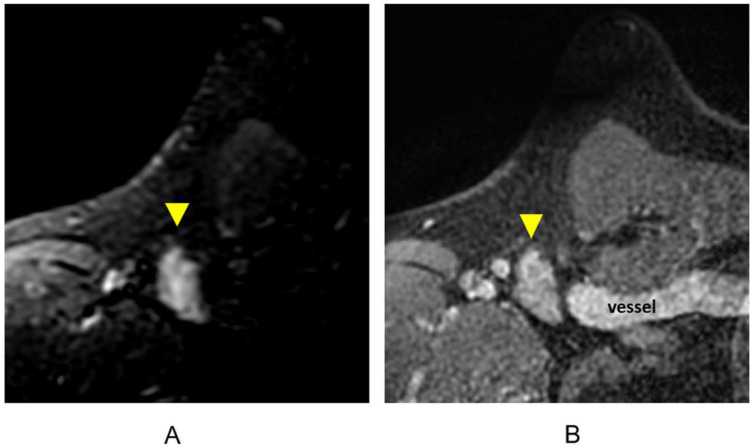
Lymph node (arrowheads). (**A**) Axial STIR sequence; (**B**) axial post-contrast T1-weighted fat-suppressed sequence.

The cortex of a lymph node has decreased signal intensity on T1-weighted sequences and increased signal intensity on T2-weighted and STIR sequences [36]. Both benign and metastatic lymph nodes can enhance rapidly and homogeneously with washout kinetics at DCE MR imaging [51,52,53]. Although lymph nodes are hyperintense on a T2 or STIR sequence (Figure 10A), lymph node morphology and margins may be characterized on the post-contrast T1-weighted fat-suppressed sequences (Figure 10B). The post-contrast T1-weighted sequences will also depict muscles, vessels, and other anatomic landmarks.

### 2.5. Breast Imaging Reporting and Data System 

The Breast Imaging Reporting and Data System (BI-RADS) was designed by the American College of Radiology (ACR) to standardize breast imaging reporting, with the goal of facilitating communication between radiologists, referring physicians, and patients. The use of standardized BI-RADS terminology is required in breast imaging reports and allows for clear and concise communication of assessment and management recommendations. A final BI-RADS assessment with an accompanying management recommendation should be given for all breast imaging studies performed. The final BI-RADS assessment categories are displayed in Table 2.

## 3. Clinical Utility of MRI for Breast Cancer

There are significant advantages and some limitations to MRI in the detection, diagnosis and assessment of treatment response to systemic therapy as summarized in Table 3 and outlined in the below scections.

### 3.1. Identification of Cancer in the Breast and Axilla on MRI

#### 3.1.1. Tumor Size Estimation and Factors Contributing to MRI-Pathology Concordance

Several studies have demonstrated that breast MRI is the most accurate imaging modality in tumor size estimation and extent of disease [55,56,57,58,59]. For instance, one study reported that tumor size on pathology was not significantly different from that seen at MR imaging but was underestimated by 14% at mammography and 18% on US [56]. That said, some studies have shown that MRI can overestimate tumor size in 9–37% of cases and also underestimate tumor size in 5–18% of cases [60,61,62,63]. Certain factors have been identified as influencing MRI–pathology size discordance, such as the presence of NME or DCIS, which can result in overestimation of tumor size by MRI [60,61,62]. 

#### 3.1.2. Evaluation of the Pectoralis Musculature, Chest Wall, and Nipple–Areolar Complex

The involvement of the pectoralis major or minor muscle alone is not considered chest wall involvement by American Joint Committee on Cancer staging but can impact surgical planning. MRI has been shown to reliably demonstrate pectoralis muscle and chest wall invasion, as indicated by abnormal enhancement of these structures [64,65]. In the absence of muscle enhancement, obliteration of the fat plane between the tumor and muscle does not indicate muscle involvement [64,65]. MRI is also able to detect malignant invasion of the nipple–areolar complex with a reported sensitivity of 94% and a specificity of 86% [66], which can aid in patient selection for nipple-sparing mastectomy. 

#### 3.1.3. Multifocal/Multicentric Disease 

Pre-operative breast MRI is particularly useful in the detection of multifocal and multicentric disease, as multifocality/multicentricity is often underestimated in both mammography and US [67]. A meta-analysis evaluating 19 studies demonstrated that MRI detects additional multicentric or multifocal disease in 16% of women with breast cancer [68]. This study found that for every three women with additional suspicious MRI findings, approximately two will have cancer and one will have a false-positive finding (TP:FP ratio = 1.9). This study also demonstrated a conversion from wide local excision (WLE) to mastectomy or to more extensive surgery in 8.1% and 11.3% of multifocal/multicentric cases, respectively. Further, in women who did not have additional malignancy on final histology, MRI-detected lesions resulted in a conversion from WLE to mastectomy in 1.1% of cases and from WLE to more extensive surgery in 5.5% of cases [68]. This highlights the importance of obtaining biopsy proof of suspicious lesions found upon MR imaging prior to proceeding to more extensive surgery.

#### 3.1.4. Contralateral Disease

Studies have shown that breast MRI can detect a mammographically occult malignancy in the contralateral breast in 3–5% of patients with newly diagnosed breast cancer [17,69,70,71,72,73]. This also applies to women with dense breast tissue where a recently published study found the use of preoperative breast MRI reduced metachronous contralateral cancers [74].

#### 3.1.5. Evaluation of Axillary Lymph Nodes 

While US is the first-line modality for axillary imaging as it allows the visualization and image-guided sampling of abnormal level I and II axillary lymph nodes to assist in accurate pre-treatment staging [75,76], MRI is able to provide a global view of the axillae, allowing for comparison of both sides and enhancing the detection of a focally abnormal lymph node [36]. In addition, breast MRI can provide the assessment of the level III and internal mammary nodal stations.

The criteria used for sonographic evaluation of lymph nodes can also be applied at MR imaging. Specifically, the complete or partial replacement of a node with an ill-defined or irregular mass, complete or partial effacement of the fatty hilum, and abnormal morphology have been shown to have a high specificity for nodal metastasis in the setting of invasive breast cancer [36,52,77,78,79]. In a small study evaluating breast cancer patients, two additional MRI-specific nodal imaging features were reported as having potential in predicting nodal metastases–(1) “rim enhancement”, defined as increased signal intensity at the periphery of a node on DCE MR images and (2) “perifocal edema”, defined as increased T2/STIR signal in the fat surrounding a node [77]. These two findings had a 100% predictive value for the detection of axillary nodal metastases in the 56 breast cancer patients evaluated. 

Lymph node size should not be used as a criterion for assessment since the overall size of a node has poor diagnostic accuracy for predicting metastatic nodal disease on imaging modalities [36,52,53,80,81,82]. In fact, axillary lymph nodes that demonstrate an oval morphology, smooth margins, and a uniform cortical thickness measuring ≤ 3 mm have a very high negative predictive value (NPV) for excluding metastases, irrespective of overall nodal size [82,83]. Symmetric bilateral axillary lymph nodes with smooth cortices have a high NPV for excluding metastasis [77]. MRI can also be valuable in identifying lymph nodes missed by an operator-dependent US exam, as seen in one study which found that MRI detected metastatic nodal disease in 15% of patients who had false-negative axillary US [84]. Given that the modalities have similar sensitivities (US 99.1% vs. MRI 97.4%) and specificities (US 15.4% vs. MRI 15.4%), the routine use of axillary US following a normal axillary MRI is not warranted if the entire axilla is included in the imaging field of view (FOV) without obscuration by (an) artifact(s) [85]. 

#### 3.1.6. Evaluation of Internal Mammary Lymph Nodes (IMLNs)

Breast cancer involving IMLNs is associated with a worse prognosis, including decreased overall survival and higher rates of distant metastatic disease within three years following treatment [86,87,88]. IMLN metastases usually occur with concurrent axillary metastases; however, isolated IMLN metastases can occur in 1–5% of breast cancers [36,89]. Tumors with involved IMLNs are characteristically associated with a deep or medial lesion [36,90]. A retrospective study showed that the MRI identification of tumor contact with an internal mammary perforator vessel had high accuracy in predicting ipsilateral and contralateral IMLN involvement [91]. As the surgical dissection of IMLN metastases has not shown a survival benefit, surgical treatment of IMLN metastases is not typically performed, particularly given the associated morbidity [90]. Regional nodal irradiation that includes the IMLNs has demonstrated a 10-year reduction in distant metastases and improvement in disease-free survival [92,93]. For this reason, it is essential that involved IMLNs are identified at the time of staging imaging. Despite this, no clear guidelines or established size criteria exist for IMLN evaluation and reporting. 

In studies assessing the presence of IMLNs in asymptomatic high-risk women presenting for screening breast MRI, normal IMLNs measuring 2–10 mm were detected in approximately half of the women screened [94,95]. In the setting of a recently diagnosed breast malignancy, however, subcentimeter IMLNs can contain metastatic disease [88,89,96,97]. The level of clinical suspicion is higher when the primary tumor is located in the deep or medial aspect of the breast. In equivocal cases, further evaluation with PET/CT can be helpful as studies have demonstrated that PET/CT is superior to conventional imaging for the detection of IMLN metastases [98,99,100,101].

### 3.2. The Impact of MRI on Decisions for Breast Conservation Therapy

#### 3.2.1. Breast Conservation Therapy

Breast conservation therapy (BCT) with lumpectomy and whole-breast radiation therapy is a well-established treatment for women with early-stage breast cancer. The primary objective of BCT is to achieve equivalent outcomes as mastectomy while allowing women to maintain a cosmetically acceptable and sensate breast. Whole-breast radiation therapy after breast-conserving surgery (BCS) is meant to eradicate potential microscopic residual disease, which can lead to approximately a 20–60% recurrence rate, with risk of recurrence influenced by patient and tumor characteristics such as age, tumor grade, tumor size, and hormone receptor status [102]. While the absolute benefit of radiation after BCS varies according to patient and tumor characteristics, the proportional decrease in disease recurrence and reduction in breast cancer death varies little [102]. Several prospective randomized trials have shown long-term equivalence between BCT and mastectomy in terms of local control and overall survival [103,104]. More recent population-based studies meant to reflect use of more modern therapies have continued to support the use of breast conservation therapy compared to mastectomy [105,106]. 

Historically, adjuvant whole-breast radiotherapy has been delivered over the course of 5 weeks. However, a 3-week hypofractionated course has been encouraged in light of studies showing similar tumor control and cosmetic outcome between the regimens [107,108]. An even shorter treatment alternative, whole-breast radiotherapy for women with early stage 1 hormone-sensitive low-risk breast cancer, is accelerated partial breast irradiation (APBI). APBI targets the lumpectomy cavity (LC) with a margin while largely sparing normal surrounding breast tissue from high-dose radiation. APBI takes advantage of the fact that ipsilateral breast tumor recurrence after BCS occurs at or near the location of the initial breast cancer in approximately 75–90% of cases regardless of whether the patient receives adjuvant whole-breast radiation [109]. APBI can be delivered using external bream radiotherapy, low- or high-dose rate brachytherapy, and intraoperative radiotherapy. The 10-year results of NRG NSABP B39-RTOG 0413 and the RAPID trial reported a difference in local control between whole breast radiotherapy and APBI of < 1% [110,111]. The most recently published APBI guidelines from ASTRO expanded the criteria to include more women, such as those with low-risk DCIS, as defined in RTOG 9804 and women as young as 50 years old [59]. With growing data showing excellent ipsilateral breast tumor control in a larger patient population, it is reasonable to expect an increase in the utilization of APBI within the field of breast radiotherapy. 

#### 3.2.2. The Use of MRI for Preoperative Staging and Surgical Planning

Despite the enhanced sensitivity of breast MRI, the routine use of breast MRI for preoperative staging remains controversial. MRI has been shown to change surgical management most often to a more radical surgery without an indication that it improves surgical care or prognosis [112,113]. Two randomized trials, the United Kingdom COMICE trial and the Dutch MONET trial demonstrated that preoperative MRI did not reduce re-excision rates for BCS [22,114]. Furthermore, a meta-analysis of four studies including 3169 patients demonstrated that preoperative MRI did not impact the risk of local recurrence (LR) or recurrence-free survival (RFS) at 8 years [115]. In contrast, other literature supports the idea that MRI reduces re-operation rates [116,117,118,119]. Additionally, a recently published study evaluating 261 patients showed that preoperative breast MRI was associated with reduced LR in women with dense breast tissue, suggesting that this subset of patients may in fact benefit from a preoperative MRI [74]. The results of the ongoing Alliance A011104/ACRIN 6694 trial, which aims to compare the 5-year local-regional recurrence rate following BCT in women at high risk of LR, may shed some light on the relationship between preoperative breast MRI, surgical outcomes, and costs. 

#### 3.2.3. Assessment of Tumor Response after Neoadjuvant Therapy 

Neoadjuvant therapy (NAT) can allow more patients to undergo breast conservation by reducing the primary tumor size in patients that otherwise would have been ineligible [120,121], and MRI has a significant role in assessing the response to therapy. Several factors have been shown to affect the predictive value and diagnostic accuracy of MR imaging in the assessment of treatment response to NAT, including tumor molecular subtype, tumor imaging phenotype, the chemotherapeutic regimen, and the pathology response criteria utilized for assessment. The most common classification of a pCR is the resolution of all invasive disease with or without a residual in situ disease in the breast and clearance of axillary nodal disease. 

Breast MRI is currently the most accurate imaging modality for the assessment of tumor response to NAT [57,58,122,123,124,125]. Overall, the published literature shows good concordance between residual tumor size measured on MRI and tumor size on pathology following surgical excision, particularly when predicting the presence of residual disease at final pathologic examination. Breast MRI to evaluate response to NAT is one of the recommended clinical indications by the American College of Radiology (ACR) and European Society of Breast Imaging, and also in the NCCN guidelines as an optional tool [126,127,128]. The use of contrast-enhanced breast MRI to determine disease response has a sensitivity approaching 90%, a specificity of 60% to 100%, and an accuracy of approximately 91% [57,58,59,70,123,129,130,131,132]. While the PPV of breast MRI following NAT is high at 93%, the NPV is only moderate at 65%, which decreases the overall diagnostic accuracy of breast MRI to 84% [122]. As such, a complete imaging response at breast MRI is not sufficient to allow patients to forgo surgical excision. 

#### 3.2.4. MR Imaging Response by Molecular Subtype 

Published studies reveal that women who exhibit a pCR to neoadjuvant chemotherapy demonstrate improved disease-free survival [133,134,135,136,137]. The independent association between triple negative/HER2-positive molecular subtype and pCR is well documented [138,139]. Interestingly, breast CE-MRI has greater accuracy in assessing residual tumor size following NAT in triple-negative and HER2-positive/ER-negative tumors [140,141]. These tumors commonly demonstrate concentric shrinkage on post-NAT MR evaluation, and residual disease is easier to measure and quantify. MRI is less accurate in the evaluation of residual disease in luminal subtypes [85,86,135,136,137]. HER2-negative luminal breast cancers are more likely to show residual disease as small foci or scattered cells at the tumor bed (tumor fragmentation) which can often result in an underestimation of residual disease extent at MR imaging [142].

#### 3.2.5. MR Imaging Response by Imaging Phenotype

Several studies have shown that kinetic changes in tumor enhancement on MR imaging during NAT are identified prior to changes in tumor volume [143,144,145]. Imaging phenotypes of breast cancer affect the reliability of breast MRI in the assessment of tumor size and NAT response. For example, MRI can more accurately predict residual disease on post-treatment imaging if the breast cancer initially presents as a mass that has clear margins for measurement or if the mass is more enhanced than the BPE [146]. Breast cancers that present as masses with clearly defined margins tend to demonstrate concentric shrinkage on post-NAT MR imaging, permitting more accurate size measurements. In contrast, breast cancers presenting as NME on pre-treatment MRI are more likely to show residual disease as scattered cells or small foci at the tumor bed on post-NAT MR imaging [146]. This type of response is known as tumor fragmentation and can decrease the accuracy of MRI in predicting residual disease resulting in the underestimation of disease [146,147,148,149,150]. Conversely, MR imaging may overestimate residual disease in cases where reactive inflammation and/or fibrosis are present at the treated tumor bed [150,151]. 

#### 3.2.6. MRI in Assessing Nodal Response to NAT

The MRI of the axilla is only 61.0% sensitive for the detection of residual nodal disease after neoadjuvant chemotherapy [152,153]. In comparison, the reported sensitivities of axillary US and FDG PET/CT for detection of residual nodal disease are 69.8% and 63.2%, respectively [152,154]. As no imaging test can yet reliably detect residual nodal disease, a surgical axillary assessment remains warranted following the completion of NAT. 

#### 3.2.7. Systemic Therapy

Certain chemotherapeutic regimens can impact the diagnostic accuracy of breast MRI. Specifically, taxane-based therapies, antiangiogenic agents, and ER modulators may alter perfusion to the breast. It is hypothesized that anti-vascular effects impact contrast enhancement [146,151]. In these cases, residual disease may be underestimated [146,151]. 

#### 3.2.8. MRI vs. FDG PET/CT in Assessment of NAT Response

A meta-analysis of 595 patients from 10 studies evaluated the accuracy of MR imaging versus FDG PET/CT imaging in measuring responses to NAT [125]. The study found that the timing of imaging influenced diagnostic accuracy. MRI outperformed FDG PET/CT after the completion of neoadjuvant therapy through its higher sensitivity. However, FDG PET/CT outperformed MRI during neoadjuvant therapy through its higher specificity [125]. This suggests that although MRI may be the superior modality at assessing residual disease after the completion of NAT, FDG PET/CT can be considered when assessing response during therapy in select clinical scenarios.

### 3.3. The Role of MRI in Assisting with APBI Candidacy

#### MRI-Based Eligibility for APBI

A meta-analysis of six studies evaluated patient eligibility for ABPI utilizing MRI, in which 6–25% of patients were deemed ineligible, with a pooled event rate of 11% (95% CI: 6–19%) [155]. In this study, the addition of breast MRI reduced inter-study heterogeneity and found that women who were premenopausal, at least pT2, or with invasive lobular carcinoma (ILC) were more likely found to be poor candidates for APBI. Similarly, in a mixed retrospective and prospective study of 521 women who underwent physical exam and mammography with or without US, MRI changed eligibility in 12.9% of patients [156]. In this study, univariate analysis indicated that tumor size ≥ 2 cm, age < 50, diagnosis of ILC, and HER2 positivity were associated with a higher risk of ineligibility after MRI. A scoring index was generated correlating the presence of 0, 1, 2, or 3 of these risk factors with a 2.8%, 13.2%, 38.1%, and 100% risk of MRI identifying occult disease, respectively. Additionally, a retrospective study conducted on a cohort of mainly young or high-risk patients found a PBI ineligibility rate of 38%, mainly due to the presence of multicentric and contralateral disease [157]. Current indications for APBI include those that are low-risk DCIS or stage 1 hormone-sensitive breast cancer in women older than 50 years of age. MRI was not utilized in 3 randomized trials evaluating APBI, which demonstrated that in-breast recurrence was non-inferior to that achieved with whole-breast radiation and event rates of in-breast recurrence were very low in both arms (RAPID, NSABP B39/RTOG 0413, University of Florence). Therefore, the potential utility of MRI for evaluating for APBI eligibility so far is mostly in higher-risk populations for whom APBI is not standardly indicated. 

Preoperative MRI may be most useful for women who appear eligible for APBI by less selective APBI guidelines [158], such as those from Européen de Curiethérapie-European Society for Therapeutic Radiology and Oncology (GEC-ESTRO) [158]. MRI-based APBI screening may find that the highest utility in efforts aiming to evaluate a broader patient population and less clinically relevant when evaluating a very low-risk subgroup.

### 3.4. Use of MRI for Post Breast Conservation Therapy Evaluation

Fat necrosis is commonly encountered following BCT and can present as a wide range of imaging findings on MRI. Enhancements related to fat necrosis should have an associated fat signal and may present as an oil cyst, a rim-enhancing mass, an area of focal enhancement, diffuse enhancement, distortion, or irregular masses. If the associated fat signal is not apparent, the MR imaging appearance may mimic a malignancy, prompting an image-guided biopsy [159]. 

Following breast radiation therapy, skin and trabecular thickening and edema are expected and present as increased T2/STIR signal in the skin and breast parenchyma. These findings gradually decrease over time but may never entirely resolve. In fact, edema in the post-treated breast can be seen in more than 25% of women six years following radiation therapy [47]. Comparison with prior studies is critical, as breast and skin edema following radiation therapy is expected to decrease or remain stable over time and increasing edema can be a sign of recurrent disease.

**Table 3:** Strengths and Limitations of MRI.

Strengths	Limitations
High sensitivity [5,6,7,8,9,10]	Cost
Tumor size estimation [55,56,57,58,59]	Accessibility
Defining extent of disease	Claustophobia
Pectoralis major/minor invasion [64,65]	IV gadolinium contrast allergy (rare)
Invasion into the nipple–areolar complex [66]	Presence of a MRI incompatible implantable device
Multicentric/multifocal disease [68]	
Mammographically occult disease in contralateral breast [17,69,70,71,72,73,74]	
Metastatic involvement of axillary or IMN nodes [36,52,77,78,79,91,96,97]	
Tumor response to NAT [57,58,59,70,120,121,122,123,127,128,129,130]	
Post treatment changes in the breast	
Non-ionizing radiation	

## 4. Breast MRI for Radiation Treatment Planning 

### 4.1. Identification and Sequences for Cavity and Clips 

#### 4.1.1. Identifying Seromas and Surgical Cavities 

**Figure 11:**
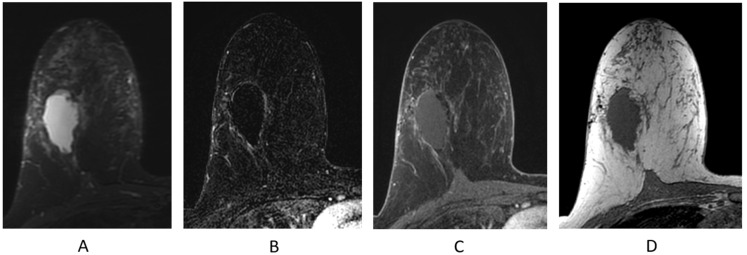
Seroma/surgical cavity on breast MRI. (**A**) Axial STIR sequence; (**B**) axial post-contrast subtraction; (**C**) axial post-contrast T1-weighted fat-suppressed sequence; (**D**) axial T1-weighted non-fat-suppressed sequence.

The normal appearance of the breast following partial mastectomy often includes a seroma at the surgical site (Figure 11). Seromas and surgical cavities containing fluid will demonstrate increased T2/STIR signal and be most conspicuous on a fluid-sensitive sequence (Figure 11A). Seromas are T1 hypointense (Figure 11D) and typically non-enhancing, but can demonstrate thin, smooth, rim enhancement on the T1-weighted post-contrast sequences (Figure 11B,C). Most seromas slowly decrease in size over time and resolve, leaving a residual non-enhancing scar on breast MR imaging. 

#### 4.1.2. Identifying Surgical/Biopsy Clips

**Figure 12:**
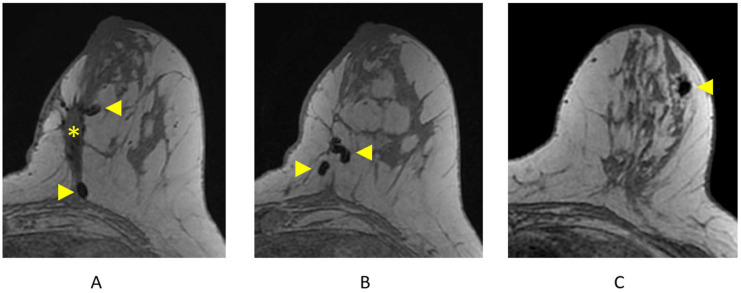
Surgical and biopsy clips on axial T1-weighted breast MRI. (**A**) Lumpectomy site (asterisk) and areas of focal susceptibility representing surgical clips (arrowheads); (**B**) additional surgical clips (arrowheads); (**C**) focal susceptibility artifact representing a breast biopsy clip/tissue marker (arrowhead).

Clips are best visualized on the pre-contrast T1-weighted non-fat-suppressed sequence and are represented as areas of focal susceptibility artifact (“signal void”) (Figure 12). Careful correlation with recent mammography is important to confirm the presence of surgical or biopsy clips, as focal areas of susceptibility artifact may also represent other processes, including hemosiderin deposition.

### 4.2. Treatment Planning Position and Co-Registration 

#### 4.2.1. CT Simulation Position

Breast MRI is not being utilized in routine clinical practice for breast radiotherapy treatment planning but has been investigated in research protocols. One challenge for the routine use of MRI in radiation planning is patient positioning. Breast radiotherapy planning and treatment have been traditionally performed in the supine position with arms overhead, while a clinical breast MRI is acquired in the prone position. Breast MRI in the supine position has been shown to be feasible with thoughtful consideration of imaging coil position, imaging parameters, and motion compensation techniques [25,160,161]. These studies show that supine MRI can have comparable imaging quality; thus, there may be potential future implications in surgical and radiation planning where patients are routinely supine. 

A more widely used alternative to supine breast MRI for radiation planning is to perform CT simulation in the prone position. Breast radiotherapy in the prone position is being more commonly utilized and has been shown to have benefits, particularly for women with larger and/or pendulous breast and left-sided breast cancer [162,163,164,165]. The boundaries of the breast CTV volume will vary to some degree between the supine and prone positions. Due to variances in body habitus, breast density, and breast size, the breasts may naturally lie more laterally or inferiorly when supine, which can make it more challenging to limit the dose to organs inside the thoracic cavity compared to a prone setup. The prone position has several advantages for radiation delivery, including a reduction in respiratory motioning, displacing the LC and breast tissue farther from the heart and lungs and improving dose homogeneity throughout the breast [166,167]. Treatment in the prone position can more easily incorporate standard diagnostic MRI with the co-registration of imaging to aid in target and organ-at-risk (OAR) delineation. 

#### 4.2.2. CT-MRI Co-Registration

In the process of co-registration, sometimes incorrectly referred to as a fusion, the CT images serve as the primary dataset, and the MR images are matched to the anatomy of the primary data set. Anatomical registration points are used to align the images, such as the nipple, sternal notch, chest wall/ribs, and tip of the scapula. Further refining of the co-registration can occur by aligning the vasculature and breast parenchymal markings, particularly in the area of the LC, given that this is the high-risk target. To reduce co-registration errors, the patient should be scanned in as close to the same position as possible. This includes arm positioning, which can affect breast position and contour. A patient’s arms are positioned overhead for radiation planning, but positioning can vary for a standard diagnostic MRI. Specifically, routine arm position for MRI may differ by institutional protocol and optimal positioning can vary depending on the coil manufacturer or model. Further, MRI with arms down may be preferred in some cases, such as a smaller patient where arms overhead lead to less breast tissue in the coil, or in patients with more mobile breast tissue, where an arm down may allow for a more even distribution of tissue over the image volume [168]. Given the variation in standard MRI protocols, it is important to perform MRI in the treatment with position planning or careful consideration of potential differences in the patient position if a diagnostic clinical MRI is being co-registered for planning purposes.

### 4.3. Radiation Therapy Target Volumes 

#### 4.3.1. Lumpectomy Cavity

When a well-visualized seroma is not present at the time of CT radiation treatment planning, there can be large variations in cavity delineation between observers [169,170,171]. Strategies to identify the cavity and interpret planning CT images include referring to operative notes, surgical clips or fiducials, clinical examination, and pre/postoperative imaging (mammogram, US, MRI). The use of CT findings and surgical clips to identify the tumor bed is considered the current gold standard for radiation planning [172]. Surgical clips are better visualized on CT than on MRI. This is particularly true when clips are close to the breast-tissue–chest-wall interface [173]. Not all institutions routinely place surgical clips or place them in all cases. MRI is better than CT at distinguishing postoperative changes, such as a small seroma, hemorrhage, fibrosis, or edema, from normal breast tissue. Further, CT-based imaging for LC delineation can be particularly challenging in the case of dense breasts, small cavities, and a delay from surgery to RT planning [169,171]. Several studies have explored the feasibility and potential role of MRI in boost or APBI treatment planning. 

Al-Hammadi et al. found that the use of supine breast MRI improved the visibility of the cavity and reduced interobserver variability (IOV) compared to CT [174]. Similarly, Jolicoeur et al. also noted a reduction in IOV, as well as a smaller cavity volume, with MRI compared to CT [175]. Further, Jacobsen et al. found in a series where clips were occasionally but not routinely used such that both MRI and CT had low IOV; however, T2-weighted MRI was the best at demarcating the interface between the seroma and chest wall, skin, and dense breast tissue [176]. These studies suggest that MRI may have utility in defining an LC without surgical clips. 

On the other hand, when surgical clips are present, there is a lack of evidence that MRI improves the IOV for tumor bed delineation compared to only using a CT/clip-based approach [177,178,179,180,181]. IOV was either similar using MRI compared to CT alone, or the incorporation of MRI increased variability in contours between observers. In general, there are inconsistent conclusions regarding the impact of MRI on determining the cavity volume, the cavity visibility score (as defined in Smith et al. [182]), or the centroid of the cavity [173,177,178,179,180,181]. Studies also vary in the approach to image co-registration, type of MR sequences, image quality, and observer knowledge of MRI and/or breast radiotherapy. Study outcomes are also likely impacted by MRI timing after surgery, which is a factor in clip migration, seroma absorption, and changes from scar tissue formation [183,184]. Furthermore, the type of BCS closure technique can also influence MRI interpretation, with full-thickness closure surgical techniques and oncoplastic procedures being more difficult for visualizing the LC. MRI can lead to an overestimation of the LC size as it provides better visualization of soft tissue abnormalities between clips that otherwise appear as normal breast tissue on CT [173]. 

In summary, the incorporation of MRI for cavity definition is still under investigation, and there are insufficient data to support the routine incorporation of MRI for radiation treatment planning. The existing data suggest that MRI may be the most useful in assisting with tumor bed definition when clips are not present. In general, clips are good surrogates for the LC; therefore, including MRI sequences (such as T1-weighted sequences without fat suppression or T1-weighted GRE sequences) in which clips are best visualized should be incorporated. LC delineation on MRI may be the most challenging to interpret in the setting of full-thickness closure surgery and oncoplastic procedures due to less of a clear seroma and the degree of peri-cavity postoperative changes. Despite the limitations in the existing literature, it does appear that certain clinical situations may benefit from the use of MRI to better define the LC boundaries, such as cavities that interface with an axillary seroma, where toxicity may be higher from an overestimation of the cavity size [181]. Defining each of those clinical situations that could benefit from the addition of a breast MRI requires further study. 

#### 4.3.2. CTV and PTV Expansions

The clinical target volumes (CTVs) for breast radiotherapy include the highest-risk tissue around the LC or tumor bed (lumpectomy CTV) and the breast tissue (breast CTV). The lumpectomy CTV is typically a 10–15 mm expansion on the LC/tumor bed volume and is limited posteriorly similar to the breast CTV. Given the size of this expansion, large variations in the LC volume will be more clinically relevant than small ones. The breast CTV is defined by the glandular and fatty breast tissue seen on CT and the radio-opaque marker demarcating palpable breast tissue placed by the radiation oncologist at the time of CT simulation. The cranial, medial, and lateral borders of the breast are highly variable depending on breast size, patient position, and breast ptosis. Medially, the breast CTV should not cross the midline, and the posterior border excludes the pectoralis and serratus muscles unless clinically warranted by surgical pathology (RTOG Contouring consensus; MA 39 and NSABP B51 protocols). The planning target volume (PTV) is generally a 5–7 mm expansion of each CTV and generally should not cross the midline. The CTV and PTV volumes are limited to 5 mm from the skin surface to account for the dose build-up region.

#### 4.3.3. The Impact of MRI on Whole-Breast Radiotherapy Planning

The role of MR imaging in delineating the breast CTV for radiation planning has been evaluated in several small series. Giezen et al. found that supine MRI-based volumes led to increased glandular breast tissue volumes by 4%, due to an extension of breast tissue further in the cranio-lateral and cranio-medial directions on MRI compared to CT [185]. Pogson et al. also noted a larger breast CTV in the prone vs. supine position [178]. In both studies, IOV by imaging modality was similar. Dundas et al. showed that while whole-breast CTV volumes were smaller on supine vs. prone CT when using MRI, the ultimately target volumes were the same after the CTV was expanded to a PTV and cropped 5 mm inside of the external skin contour [186]. Similarly, Kirby et al. found minimal differences in CT vs. MRI-based breast target volumes when expansion for PTV was taken into account [173]. In summary, MRI does not appear to offer significant advantage over CT for defining whole-breast targets for whole-breast radiotherapy.

#### 4.3.4. Preoperative APBI 

Preoperative APBI offers the potential advantages of better target visualization given intact tumor and smaller treatment volumes prior to resection, allowing for greer sparing of uninvolved breast tissue. The correlation between increased partial breast treatment volumes and poor cosmetic outcome makes strategies for limiting the target volume appealing [187,188]. This may be of further interest with the increasing use of oncoplastic procedures, which tend to result in larger post lumpectomy treatment volumes. Additionally, smaller treatment volumes and a strong correlation with pathological tumor size seen on MRI also facilitate dose escalation to a geometrically accurate target [189]. A disadvantage of preoperative APBI, on the other hand, is that the axillary nodal status in unknown, so careful examination of the axilla is recommended. Preoperative APBI delivered by using conformal external beam techniques or stereotactic body radiotherapy (SBRT or SABR) have been explored with and without MRI for treatment planning on a number of small prospective trials [126,190,191,192,193,194,195,196,197].

Optimal MRI sequences for treatment planning in the preoperative setting are CE T1-weighted fat-suppressed sequences for tumor visualization and T2-weighted sequences to differentiate from post-biopsy changes. An advantage of the presence of gross tumor is the visibility on MRI, which can reduce the IOV seen in the postoperative setting. As such, most studies have considered MRI critical for target definition given that not all breast tumors are reliably identified on CT alone and MRI better demonstrates tumor spiculation/irregularity [192,198]. While MRI offers more detailed anatomy, extended tumor spiculae, markers within tumors, blood vessels near tumors, and areas of increased enhancement in glandular breast tissue can lead to increased IOV in tumor delineation [199]. In an effort to reduce the IOV in target volume definition and aid in more comparable outcomes between future clinical trials, consensus-based guidelines for contouring primary breast tumors on MRI in preoperative PBI trials have been developed [199]. 

It has been hypothesized that definitive ablative radiotherapy could potentially offer an alternative, non-invasive curative approach for patients with early-stage breast cancer. Achieving a pCR with preoperative radiotherapy with or without systemic therapy would introduce the possibility of eliminating surgical resection for early-stage breast cancer. In a systematic review evaluating response and clinical outcomes after preoperative APBI followed by BCS in patients with low-risk breast cancer, recurrence rates were overall low, and three out of six studies reported pCR rates ranging from 9–48% [200]. Pathologic CR rates increased with increasing duration between preoperative radiotherapy and surgical excision. The ROCK trial demonstrated a 9% pCR rate 2 weeks after 21 Gy in a single fraction using Cyberknife in 22 patients [201]. Nicholas et al. showed a 15% pCR rate in 27 patients 3 weeks after 38.5 Gy in 10 fractions delivered twice daily [196]. The highest rates were found in the ABLATIVE trial with a pCR in 33% and 48% of patients 6 and 8 months after 21 Gy in a single fraction, respectively [194]. A preoperative paradigm has been shown to be feasible and can achieve acceptable toxicity and cosmesis with good local control on short-term follow-up; however, larger randomized trials with longer follow-up and larger patient numbers are needed. 

Preoperative breast radiotherapy studies also offer a means for assessing the relationship between dose, tumor characteristics, and tumor response rates [194,202]. Quantitative and semi-quantitative analysis of pre- and post-treatment MRI has the potential to create biological imaging correlates and identify predictors of treatment response. This would facilitate appropriate patient selection for preoperative therapy, as well as broader implications for a more personalized medicine-based approach. 

## 5. MRI-Guided Breast Radiotherapy 

### 5.1. MRI-Linac Systems

MR-guided radiotherapy utilizing hybrid MRI-linac (MRL) systems is beginning to take a clinical foothold with its novel ability to provide real-time data on target cavity position and offer daily dosimetry optimization. The MRL combines an MRI scanner, with differing Tesla strength depending on themanufacturer, and an MV-energy photon linac mounted on a ring-shaped gantry, which enables real-time MRI acquisition during radiotherapy to account for anatomical position and motion [203]. Respiratory gating can be performed so that the radiation beam turns off if the target moves out of the pre-specified window [204]. Alternatively, target tracking entails the changing of a beam shape according to the target motion which is detected in a fast feedback loop [205,206]. These techniques can lead to the minimization and possible elimination of planning target volumes, thereby limiting normal tissue irradiation and allowing further dose escalation to target volumes. 

### 5.2. Implications of Intrafraction Monitoring

Intrafraction motion during a diagnostic breast MRI is generally limited to <3 mm, with the exception of rare large body shifts due to setup or deep breaths over 1 cm [207]. Limited intrafraction motion and enhanced visualization of the surgical cavity afforded by MRI have led some investigators to evaluate the reduction of GTV to PTV expansion for APBI from the standard 2–2.5 cm to a 1 cm expansion with MR guidance, which is more consistent with an expansion utilized in brachytherapy planning [208]. The feasibility of reducing or eliminating the PTV expansion for MRL-based APBI has been evaluated in a 30-patient prospective registry study [209]. The lack of PTV resulted in treatment volumes 52% less than the conventional 1 cm CT-based PTV and appeared to be reasonable in most patients with a mean LC displacement (AP and SI directions) of 0.6 mm. However, the largest intrafraction displacement was 6 mm, suggesting that some patients will still benefit from a PTV, and perhaps the cine MR images at the time of simulation can be used to determine PTV expansion [209]. While intrafraction motion is generally small and MRL-guided therapy may allow for smaller PTV volumes, onboard MR image guidance is important due to the heterogeneity of intrafraction and interfraction motion between patients.

Another strategy for motion management that can account for day-to-day anatomical variation is MR-based adaptive planning. Ng et al. demonstrated the feasibility of a real-time MR-guided PBI adaptive planning workflow that incorporates same-day contouring based on the MRI images acquired before treatment and same-day dose optimization with a new tangential intensity-modulated radiotherapy plan [210]. This approach is one way to help mitigate the challenges of daily setup uncertainty and better account for potential changes in the surgical cavity, breast tissue, or cardiac positioning during treatment. An important consideration for this strategy is that more time is required than standard CT-guided treatment, and it requires the presence of a clinician, dosimetrist, physicist, and therapists, making it more resource-intensive.

### 5.3. Dosimetric Impact of the Magnetic Field on Breast Radiotherapy 

One concern about using MRL for breast radiotherapy is the potential effects of the magnetic field on secondary electrons generated by ionizing radiation and their subsequent impact on skin dose. The magnetic field causes a reduction in the build-up distance due to a curved electron path trajectory, which results in a decreased entrance depth in tissue [211]. Additionally, electrons exiting the body are subjected to a phenomenon termed the electron return effect (ERE), where electrons take a circular path in the air and reenter the tissue-depositing dose at tissue–air interfaces [211,212]. The ERE dose increases with increasing magnetic strength and as such lateral ERE is strongest for a 3T and shows no effect at 0.2T [213]. While diagnostic imaging is performed with a 1.5T or 3T MRI, the use of weaker-strength magnetic fields such as a 0.35T MRI has been used successfully to visualize and track volumes during APBI [209]. ERE can be minimized when using multiple beams such as with intensity-modulated radiotherapy (IMRT); however, breast radiotherapy most commonly employs 3D conformal techniques where opposed oblique fields cover a large superficial area. This has led to some investigators lending caution to the use of MRL in whole breast irradiation (WBI). Lastly, the electron stream effect (ESE) has also been described, where electrons generated in the body start spiraling along the magnetic field towards the head and arm. One way to manage ESE is to shield areas outside of the treatment field with the use of bolus to avoid unwanted doses to the skin outside of the field [214]. 

These dosimetric concerns regarding local skin dose may be minimalized in APBI where treatment volumes are small, resulting in less skin involvement. For example, van Heijst et al. compared a seven-field IMRT APBI, a seven-field IMRT WBI and a two-field tangent WBI plans in varying magnetic strengths (0T, 0.35T and 1.5T) and found an increased skin dose with the whole breast plans that was negligible with APBI [215]. Another small study of palliative patients with intact tumors in close proximity to skin explored the impact of the magnetic field for different geometric beam arrangements (2–3 tangent beam arrangement, 5-beam IMRT and VMAT) used to deliver hypofractionated APBI (40 Gy in 5 fractions) on a 1.5T MRL [216]. In their APBI analysis, the largest variability between the plan configurations were in skin dose (more noticeable for the 3 mm than the 5 mm skin thickness), which was mitigated by adding additional beam angles. Differences seen between studies may be reflected in the difference in the number of beams, the skin thickness used for analysis or treatment planning systems [216]. Additionally, as noted by Kim et al., heart and lung doses were not significantly affected by the magnetic field, although the lung maximum was higher in the magnetic field, possibly due to the tissue–air interface at the chest wall. For higher ablative doses, caution should be taken to avoid overdosing the chest wall, which would increase the risk of chest wall pain or rib fracture [217]. While excess skin dose with the use of the MRL should be minimized for standard breast patients, this feature may be worth further investigation for those patients with cutaneous involvement who may benefit from “magnetic bolusing” [216]. 

### 5.4. Patient Position Considerations

The MRI bore is smaller than the CT bore, which can limit patient positioning options. In the supine position, he arm position may be more limited by the need to reduce the elbow span to accommodate the bore size. There is a limitation in the degree of inclination that is feasible, and a small wedge or no inclination may work best [218]. An anterior receiver coil can cause breast deformation while in the supine position, but this can be mitigated with the support of a coil bridge [160,175,181]. In the prone position, patient selection is limited by the space needed for a more pendulous breast to hang without touching the table top and for th placement of a receiver coil to the back of a patient so that a full body contour can be obtained for treatment planning. An addition consideration is patient positioning devices, which are not universally MRI-compatible, so careful selection is required and certain devices may need to be created if not commercially available.

## 6. Conclusions and Future Directions 

In the assessment and treatment of breast cancer, the use of MR imaging has been primarily used to define the extent of initial and residual disease. However, its role is evolving to offer prognostic value and help guide treatment, particularly for APBI techniques. With further study, it may eventually be feasible to omit surgical resection in some patients who demonstrate a pCR to preoperative MRI-guided breast radiotherapy and systemic therapy alone. Another promising area of study is translational field of radiomics, which offers a means for extracting quantitative biomarkers from radiological images to provide information on the subtype, grade, heterogeneity, molecular profile, and genetic signatures. Radiomics enables the prediction of tumor biology, treatment response, and risk of recurrence. MRI-guided preoperative radiotherapy and radiomics provide a means for functional imaging analyses to better understand the radiobiology of breast cancer and facilitate the selection of patients who would be most likely to respond well to this strategy. Additionally, the preoperative radiotherapy approach also allows for the use of biopsy and lumpectomy tissue samples to investigate pre- and post-radiation gene expression [191]. The optimal use of MRI will allow for further personalization of breast cancer therapy.

## Figures and Tables

**Table 1:** Summary of common indications for breast MRI.

Indication Type	Evaluation of/for:
Screening	High-risk patients Contralateral breast in patients with newly diagnosed breast cancer
Extent of Disease	Multifocal or multicentric disease Pectoralis, chest wall, or nipple involvement Neoadjuvant therapy: prior to, during, and after therapy Residual disease following lumpectomy with positive margins
Additional Evaluation of Imaging or Clinical Findings	Detecting mammographically occult primary breast cancer in women with axillary metastases Breast cancer recurrence when clinical and mammographic/US imaging findings are inconclusive MRI-guided breast biopsy Breast implants (no IV gadolinium needed)

**Table 2:** BI-RADS final assessment categories.

Category	Assessment	Risk of Malignancy
0	Incomplete–Need Additional Imaging Evaluation	N/A
1	Negative	Essentially 0%
2	Benign	Essentially 0%
3	Probably Benign	>0% but ≤2%
4	Suspicious	>2% but <95%
5	Highly Suggestive of Malignancy	≥95%
6	Known Biopsy-Proven Malignancy	N/A

Adapted from [54].

## Abbreviations

ACR	American College of Radiology
ADC	apparent diffusion coefficient
AJCC	American Joint Committee on Cancer
APBI	accelerated partial breast irradiation
BCS	breast conserving surgery
BCT	breast conservation therapy
BI-RADS	Breast Imaging Reporting and Data System
BPE	background parenchymal enhancement
CTV	clinical target volume
DCE	dynamic contrast-enhanced
DCIS	ductal carcinoma in situ
DWI	diffusion weighted imaging
ERE	electron return effect
ESE	electron stream effect
FDG	fludeoxyglucose
FGT	fibroglandular tissue
GRE	gradient echo sequence
ILC	invasive lobular carcinoma
IMLN	internal mammary lymph node
IOV	interobserver variability
LC	lumpectomy cavity
LR	local recurrence
MBD	mammographic breast density
MIP	maximum intensity projection
MRI	Magnetic resonance imaging
MRL	MR-linac
NAC	neoadjuvant chemotherapy
NME	non-mass enhancement
NPV	negative predictive value
OAR	organ at risk
pCR	pathologic complete response
PET/CT	positron emission tomography–computed tomography
PPV	positive predictive value
PTV	planning target volume
RECIST	response evaluation criteria in solid tumors
RFS	recurrence-free survival
SBRT/SABR	stereotactic body radiotherapy
SNR	signal-to-noise ratio
STIR	Short-tau Inversion Recovery
T	Tesla
US	Ultrasound
WLE	wide local excision
